# Understanding the Factors Contributing to Suicide Among the Geriatric Population: A Narrative Review

**DOI:** 10.7759/cureus.46387

**Published:** 2023-10-02

**Authors:** Akshay R Dhole, Prithvi Petkar, Sonali G Choudhari, Harshal Mendhe

**Affiliations:** 1 Department of Community Medicine, Datta Meghe Medical College, Datta Meghe Institute of Higher Education and Research (DU), Nagpur, IND; 2 Department of Community Medicine, Datta Meghe Institute of Higher Education and Research (DU), Wardha, IND

**Keywords:** older adults, geriatric population, risk factor, self-harm, suicide

## Abstract

A critical health concern for older adults is suicide, particularly for those above the age of 60 years. Despite this, research on suicide in this age group is relatively scarce compared to studies on younger populations. This article is intended to summarize the existing literature on etiological/risk factors, including problems with one's physical and mental health, social isolation, money, and life changes like retirement and the death of a spouse and methods for preventing suicide specific to the geriatric population. We conducted a comprehensive literature search to identify the original reports and reviewed publications through various databases, including Google Scholar, PubMed, and the CDC. We gathered information on Google from reputable sources such as the WHO and the National Crime Records Bureau (NCRB). Our review found that the risk factor of suicide in the geriatric population includes physical illness, familial issues, financial issues, and hopelessness. The suicide rate for older adults declined, dropping from 16.17 per 100,000 individuals to 14.25 per 100,000 individuals aged 50 to 69 years and from 27.45 per 100,000 individuals to 24.53 per 100,000 individuals for those over 70 years. A more significant proportion of elderly suicide attempters come from rural than urban locations. Young individuals have better professional opportunities in urban areas, but older people are dispersed to underdeveloped or rural areas, where they are more likely to experience social isolation. By systematically identifying these risk factors, we can develop prevention and intervention strategies to decrease the suicide rates among the geriatric population.

## Introduction and background

Suicide is a significant issue related to mental health that can happen at any point throughout a person's lifetime. It involves intentionally ending one's life, resulting in premature death [1]. The World Health Organization (WHO) predicts that one in six individuals will be 60 or older by 2030. By 2050, there will be 1.4 billion people over 60, up from 1 billion in 2020. In 2050, around 2.1 billion individuals will be 60 or older [2]. India has a 104 million geriatric population (60+ years old), or 8.6% of the population, as of the 2011 Census [3]. As per WHO, the yearly global suicide rate of 10.5 per 100,000 persons results from almost 800,000 suicide deaths yearly [4]. Suicide among older individuals is often overlooked or disregarded compared to suicide among younger age groups, particularly young adults and adolescents, as the death of an older person is perceived to have less impact than others. In 2017, the suicide rate was 16.17 per 100,000 persons aged 50 to 69 years, while it was 27.45 per 100,000 people for those over the age of 70 years [5]. In 2019, the suicide rate for individuals aged 50 to 69 was 14.25 per 100,000, while for those aged 70 and older, it was 24.53 per 100,000 [6]. In recent decades, advances in health care, extended lifespans, and changes in social patterns have led to a significant increase in the elderly population. However, this change in demographics has not been without its challenges, and one of the most troubling issues is the worrying increase in suicide rates among older adults [7]. Those 65 years and older who have never before experienced depression are said to be suffering from late-life depression. It is specified by feelings of unhappiness resulting from various situations such as loss, unfulfilled goals, or failed relationships [8].

In the geriatric population, physical sickness and functional impairment are prevalent, resulting in loss of self-determination, loneliness, pain, increasing demand on social networks, and the emergence of depression [9]. The Biopsychosocial Aging Theory of Suicide seeks to combine key factors in biology (such as debilitating physical illness), psychology (such as depression), and social relationships (such as isolation) that contribute to the development of suicidal tendencies. In conjunction with old age, these factors can amplify feelings of hopelessness, resulting in a higher risk of suicide [10]. It can be concluded that as the age of older persons increases, there is a greater likelihood of nonviolent suicide methods being preferred by them it is based on observations from a study that compared the manner of suicide and age of elderly individuals, revealing that among those aged 60-69 years, 41.6% opted for drowning and 30.6% consumed insecticides; among those aged 70-79 years, 67.8% used hanging; and among those aged 80 years or older, 57.1% consumed insecticides [7]. Recent studies have found that depression is linked to a slower ability to process information and difficulties in working memory, which play a significant role in causing cognitive problems. It may raise the likelihood of developing Alzheimer's disease in people with depression [11]. This article aims to illustrate some of the significant risk factors associated with suicide among older adults.

## Review

Methodology

This narrative review focuses on factors contributing to suicide among older adults. For this article, to identify the original reports and reviewed publications, literature was searched using databases like Google Scholar, PubMed, and CDC. The data were collected from valid Google sites like the WHO and National Crime Record Bureau (NCRB). To locate all pertinent articles, several keywords and Medical Subject Headings (MeSH) phrases were used, which included "suicide", "self-harm", "geriatric population", and "older adults". Studies of any kind were considered if they were deemed related to the subject of our review. The excluded articles are the ones that did not mention suicide among elderly adults and the ones that were not available in the English language.

Comparison of suicidal incidence between urban-rural area

According to research by Hempstead et al. [12], older persons are more likely to have access to firearms in rural areas, which can increase the risk of successful suicide attempts because of the high lethality of these weapons. In a study comparing the risk factors for older suicide attempters to younger suicide attempters, it was discovered that there was a substantial difference between the groups, with a higher percentage of elderly suicide attempters originating from rural than from urban areas. It may be demonstrated by young people having more excellent career prospects in urban regions. In contrast, older adults depart from undeveloped or rural places, leaving them prone to social aloneness [13]. From a study on 60 older adults, there were a total of 37 cases of old-age suicide reported in urban areas, compared to 23 instances in rural regions. The suicide rate is sometimes cited as being higher in cities because of various urban stresses, including crowded living conditions and social isolation [14]. Lapierre et al. [15] brought attention to the dearth of mental health specialists in rural locations, making it difficult for elderly patients to obtain timely and effective therapy. Since fewer mental health services are available, untreated mental health problems may worsen and lead to higher suicide rates.

The sequential thought process leading to suicide among older adults

**Figure 1:**
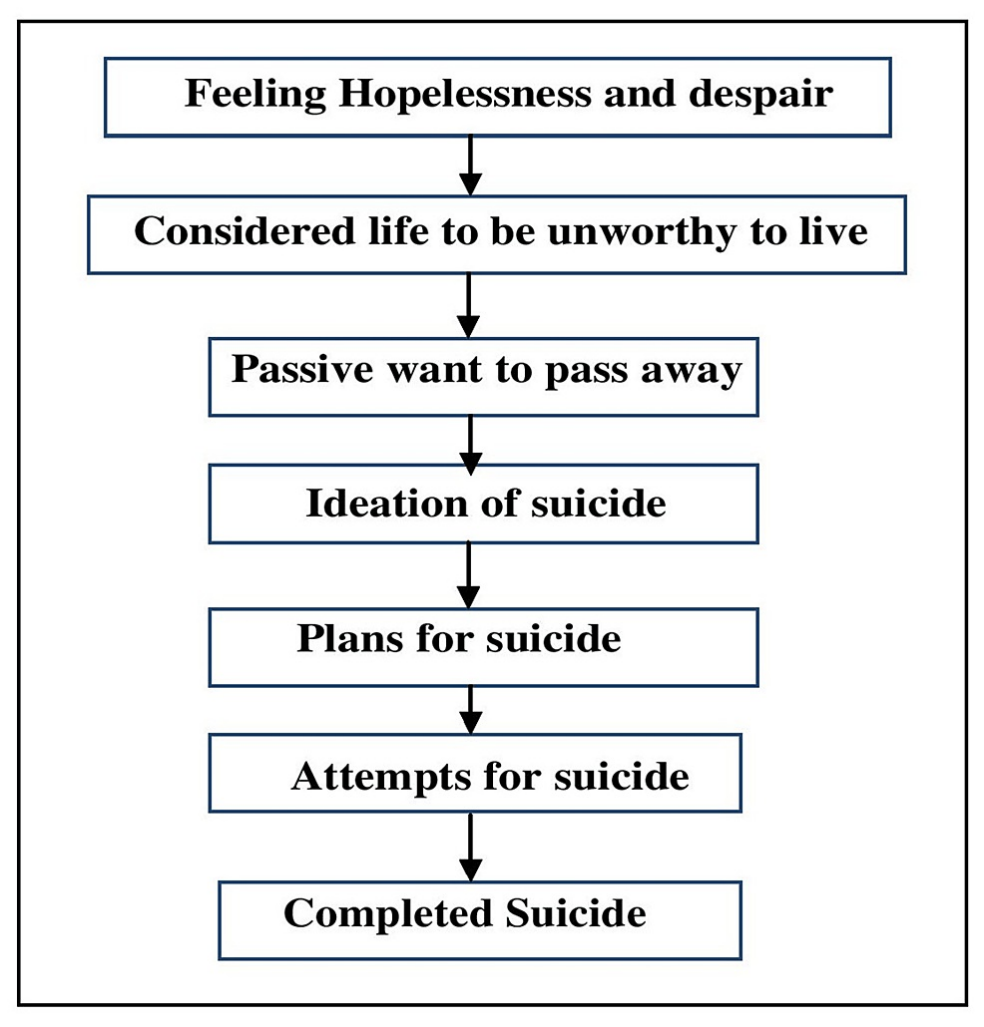
Model of suicidality

Figure 1 represents the sequential thought process that leads to suicide among older adults. First of all, the individual feels hopelessness and despair due to chronic diseases like cancer, diabetes, chronic obstructive pulmonary disease (COPD), and hypertension, which lead to physical illness. They think that life is not worth living and plan for suicide, and at a point, they complete suicide [16].

Warning signs of suicide among older adults

When concerned about the potential risk of suicide in older adults, it is crucial to remain vigilant for a range of warning signs that could indicate distress. These signs encompass a variety of behavioral, emotional, and verbal cues that might suggest the person is struggling [17]. Pay attention if they talk about feeling like a burden on others or express thoughts about isolation. Heightened anxiety levels and discussions about being trapped in unbearable emotional pain might become apparent. Additionally, an escalation in substance use could indicate their attempt to cope with their struggles. The search for access to lethal methods or discussing such means is another serious warning sign [18]. If they exhibit increased sudden shifts in mood, expressions of hopelessness and pessimism about their situation can also be concerning [19]. Additionally, it is crucial to watch for disrupted sleep patterns, either sleeping excessively or struggling with insomnia [20]. If they start talking or sharing online content about wanting to die, it is crucial to address the situation. Furthermore, the formulation of specific plans for suicide should be taken very seriously. In such cases, it is imperative to offer support and connect them with appropriate mental health resources as soon as possible, as these signs underscore a heightened risk of suicidal thoughts and behaviors [21]. Research by Pompili et al. [22] found that behavioral changes in older adults, such as withdrawal from social activities, increasing alcohol intake, or giving away possessions, may indicate suicidal intentions.

The warning signs of suicide risk in older adults can manifest in various ways, such as the individual consistently appearing sad or depressed, withdrawing from activities that once brought joy, they could also be experiencing feelings of anxiety and struggling with sleep, mood swings might become frequent and intense, neglecting personal hygiene and abandoning their physical appearance are possible signs as well as avoiding social interactions and isolating from friends and family, disproportionate feelings of guilt or shame might be expressed and a loss of interest in food could be observed. There could be a noticeable increase in the consumption of cigarettes and alcohol. Verbalizing thoughts about death, such as expressing that they have had enough or life no longer makes sense, can be concerning. An older adult who has put aside pills and other non-therapeutic drugs while unexpectedly visiting relatives and friends as if to say goodbye may be exhibiting signs of potential suicide risk [5].

Possible risk factors of suicide among older adults

**Figure 2:**
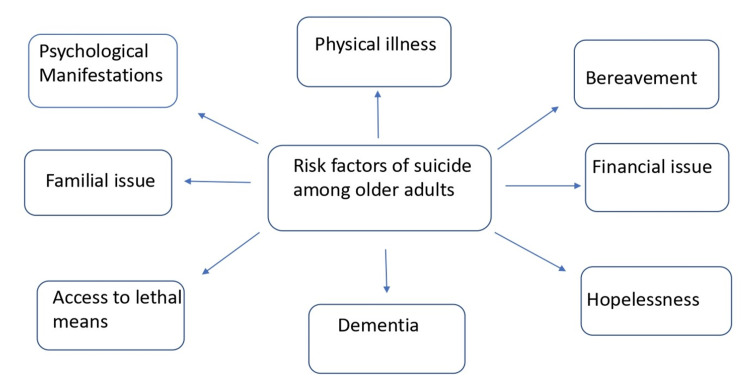
Risk factors of suicide

Figure 2 shows possible factors leading to suicide among older adults.

Psychological manifestations

In psychological autopsy investigations of senior suicides, it has been shown that 71-95% of those who lost their life by suicide had a severe psychological condition at the time of their death, and most elderly suicide studies provide clear evidence that death by suicide is significantly predicted by psychopathological issues [16]. Major depressive disorder, in particular, is frequently and significantly linked to an increased risk of suicide death. Furthermore, a strong link exists between hopelessness and a greater risk of suicide, and it is an established mediator between depression and suicide [10]. It was shown that people who died by suicide in both groups were more likely to have psychological diagnoses than controls in research on the characteristics that predict suicide among older persons compared to sudden death controls and middle-aged suicide. According to this result, mental illnesses may be crucial when identifying those with a greater suicide risk [14].

Physical illness

In addition to mental illness, functional limitations and physical infirmity raise the risk of suicide in later life. However, the prevalence of physical sickness and impairment is so high in this group that it is difficult to identify specific older adults who need assistance. For instance, record linkage studies have repeatedly discovered that people with cancer (other than common skin cancers) had about two-fold higher risk of suicide than people without cancer [9]. It is highlighted that physical problems and suicide are related, with the majority of older persons who died by suicide having underlying health ailments. Suicidal behavior in this population is strongly associated with chronic illnesses and pain, recognized as critical risk factors [23]. Suicidal behavior is related to functional limitations and certain chronic diseases, such as cancers, chronic obstructive pulmonary disease (COPD), neurological diseases, hepatic disease, arthritis, male genital difficulties, and discomfort, according to a recent study of older adults. These results imply that these health issues and functional limitations may raise the probability of suicide conduct in older people [24]. Severe pain was revealed to be a more significant etiological element for suicide than depression or any bipolar disorder in a recent population-based retrospective analysis. A strong connection between the risk of self-harm or suicide and total sickness was also noticed [25].

Bereavement

The passing of a close relative is a particularly traumatic life event. Anger, guilt, and despair are all possible emotions that can be felt throughout the mourning process, primarily when it occurs in a socially isolated setting. Studies examining the load of mourning have revealed an increased risk of adverse health consequences, including suicide, after death [26]. With variables including a lack of social integration, a poor feeling of belonging, and inadequate social support, it is thought that losing close relatives is a traumatic event linked with an increased risk of self-harm or suicide [27]. According to Mogensen et al. [28], suicide rates were most significant six months after losing a family member or close relative. The risk ratios for suicide were found to be highest among those 45 years of age and older who had lost a spouse during the preceding month. In the age range of 45-64 years old, the odds ratios (OR) of 4.33 and 3.46 were noted, respectively, in the 65 and older age range.

Familial issues

According to researchers, familial issues may be characterized as any form of instability within a family brought on by abrupt change and unrelated events that result in unstable family relationships and people who fall short of their family's expectations. Family issues were identified as the cause in about 35.0% of suicide occurrences involving older adults. Within this group, the study discovered that 18.33% had suffered abuse from family members, and 16.67% lived in poverty. Two notable cases of suicide by elderly individuals have been identified through news reports, in which abuse and poverty within their families were determined to be contributing factors [6]. A study by Van Orden et al. [29] identified perceived burdensomeness and thwarted belongingness, which can result from negative familial relationships, as significant predictors of suicidal ideation in older adults. Similarly, a study by Chen et al. [30] found that low family support and satisfaction levels were associated with increased suicide risk among older adults. According to research by Conwell et al. [31], The risk of suicide among older adults is constantly increased by familial problems such as family strife, interpersonal stress, and the death of a loved one. Family problems, such as disagreements with children or a spouse, are strongly linked to suicidal thoughts and attempts in older persons and are a key contributing factor to suicide.

Financial troubles

Financial stress is a primary risk factor for suicidal thoughts, particularly among elderly individuals who rely on steady incomes and face a challenge to cover their expenses, such as bills and food. This situation can be further exacerbated by preexisting health problems or grief, intensifying their ability to cope with these challenges. It is essential to recognize the potential risk factors and support those struggling with financial stress and its associated mental health effects [32]. In a study conducted by Luoma et al. [33], it was discovered that financial difficulties were a prevalent source of stress in the lives of older adults who committed suicide. More than 50% of the older adults who died by suicide had indicated experiencing financial problems as a stressor in the six months leading up to their deaths. This research, which involved examining the psychological backgrounds of these individuals after their deaths, also revealed that financial problems were more frequently observed among older adults who died by suicide when compared to younger adults who had the same unfortunate outcome [34]. Research conducted by Fiske et al. [35] discovered that older adults who experienced financial difficulties were more likely to be at risk of developing depression. The study involved 1,260 individuals aged 55 and older, and the results showed a notable increase in the likelihood of depression among those who reported financial strain. Another study by Amit et al. [36] explored the direct link between debt and suicidal tendencies in elderly individuals. Their research revealed that significant financial debt, encompassing credit card debt and mortgage obligations, plays a significant role in predicting both thoughts of suicide and actual suicide attempts.

Access to lethal means

Lethal means any items or methods that can cause death, such as firearms, medications, chemicals, and sharp objects. It is well-known that older adults tend to be more alone, frailer, more prone to have a plan, and more likely to utilize fatal methods when trying suicide. Hence, an older adult's suicide attempt has a higher risk of dying than that of a younger adult [37]. According to a study by Conwell et al. [38], 68% of older adults who died by suicide used a firearm. According to research, older persons are far more likely to commit suicide if they can access deadly weapons. Regardless of one's mental state, having a firearm in the house triples the chance of suicide [39].

Dementia

Extensive longitudinal research in Denmark found that people with dementia had an 8-10 times greater risk of suicide than people without dementia who were aged 50-69. However, it was found that those above 70 had a lower suicide risk [40]. Even after adjusting for mood disorder presentations, receiving a dementia diagnosis while hospitalized is linked with an elevated possibility of suicide [41]. In a Danish case-control study of people aged over 60 years who had been admitted to a psychiatric hospital and subsequently died by suicide, patients with dementia had a low risk of suicide [39]. When Koyama et al. [42] looked at 634 dementia patients, they found that 10% had suicidal thoughts. A research study found that among patients with depression, hopelessness was the most potent indicator of suicide risk over ten years. Hopelessness strongly predicted suicide intent, especially in older persons [43].

Other factors

Suicide risk is a complex and multifaceted issue influenced by various factors discussed above. Another significant factor is a history of previous adult suicidal attempts. Individuals who have attempted suicide in the past are at a heightened risk of future attempts, often due to unresolved emotional struggles. Substance abuse, particularly alcohol and drugs, also contribute to increased vulnerability. Substance abuse can impair judgment, amplify emotional distress, and lower inhibitions, making suicidal thoughts more likely to escalate into actions. [31]. Moreover, feelings of isolation and loneliness can intensify the risk of suicide. Human connections play a crucial role in mental well-being, and individuals who lack a strong support network may feel trapped in their emotional pain. A family history of affective disorders and suicide can also elevate the risk, indicating a potential genetic and environmental predisposition to mental health struggles. Certain personality traits, such as rigidity and obsession, can exacerbate vulnerability. These traits can lead to difficulties adapting to challenges and changes, heightening the emotional burden. Problems in interpersonal interactions further compound the risk, as strained relationships or social conflicts can amplify feelings of hopelessness and despair [44].

Suicide preventions strategies

A neglected fact of the mental health of geriatric suicide is mentioned earlier. Preventive measures need to be implemented so that a society free from elderly suicide is achieved [14]. Every year, on September 10, attention is drawn to the problems, reducing stigma and increasing consciousness among organizations, the government, and the general population while sending a clear message that suicide prevention is possible. World Suicide Prevention Day's triennial theme from 2021 to 2023 is "creating hope by taking action". The underlying message of this theme is to instill optimism and positivity by highlighting other options besides taking one's own life [45]. The Mental Healthcare Act, approved in 2017, assures that people who attempt suicide will get competent medical care and eliminate the criminality of suicide [46]. It is a significant step forward in treating individuals with respect and compassion. The National Mental Health Programme and Ayushman Bharat Health and Wellness Centres focus on providing top-quality treatment at the basic healthcare level. In addition, resources such as deaddiction centers and rehabilitation services are available [47]. The objective should not only be preventing suicide but also enhancing the standard of life for elderly persons [48].

Understanding the risk factors associated with suicide among older adults is essential for designing effective prevention strategies. Some key risk factors include social isolation, chronic health conditions, depression, and access to lethal means. Addressing these factors forms the foundation of any suicide prevention effort [34]. Providing information and raising awareness among older individuals about mental health, suicide prevention, and the support services accessible to them can diminish the associated social stigma and encourage them to seek assistance. It can be accomplished through community-based workshops and outreach initiatives [49]. Research supports the effectiveness of psychotherapeutic treatments, such as cognitive behavioral therapy (CBT) and dialectical behavior therapy (DBT), in reducing the risk of suicide among older individuals. These therapies help by addressing underlying psychological issues, improving coping skills, and enhancing emotional regulation, ultimately reducing the likelihood of suicide in this age group [50]. Promoting routine mental health assessments for elderly individuals can be crucial in detecting potential suicide risks. Additionally, advocating for accessible mental health resources like geriatric psychiatry facilities and telehealth services is essential [51]. Elderly individuals tend to employ highly fatal methods when attempting suicide. Implementing strategies limiting their access to firearms, medications, and other deadly means can save lives [52]. It is crucial to offer continuous assistance and follow-up healthcare to elderly individuals who have previously tried to take their own lives or are at risk, as this can significantly reduce the likelihood of them making another suicide attempt [53].

## Conclusions

In conclusion, due to the distinctive variables indicated above, the risk of suicide is high in the geriatric population, those 60 years and older. A significant cause for public concern is that focused preventative measures and treatments are required. It is essential for healthcare providers and family members to identify the warning signs in older adults and to provide support and resources to prevent a suicide attempt. To prevent elder suicide, it is critical that healthcare professionals, policymakers, and the community at significant take action. Reducing the negative perceptions associated with mental health problems, improving the availability of mental health care, managing resources effectively, and providing support are all essential steps in assisting older adults in preserving their autonomy and social relationships. By implementing a thorough and interdisciplinary plan, we can assist in decreasing the incidence of suicide rate among the geriatric population and promote healthy aging.
